# Natural Product-Induced Modulation of Androstenone Metabolism in Porcine Hepatocytes

**DOI:** 10.3390/ani15152199

**Published:** 2025-07-25

**Authors:** Christine Bone, E. James Squires

**Affiliations:** Department of Animal Biosciences, University of Guelph, Guelph, ON N1G 2W1, Canada; jsquires@uoguelph.ca

**Keywords:** boar taint, androstenone, natural products, metabolism, PXR, CAR, FXR

## Abstract

The nuclear receptors pregnane X receptor (PXR), constitutive androstane receptor (CAR), and farnesoid X receptor (FXR) regulate the hepatic metabolism of androstenone, a testicular steroid that accumulates in the fat of intact male pigs and causes boar taint. This study evaluated natural product-derived compounds and conventional agonists targeting these nuclear receptors for their effects on androstenone metabolism in primary hepatocytes from slaughter-weight boars, to assess their potential as treatments for boar taint. Cells were incubated with natural products, conventional agonists, or dimethyl sulfoxide (DMSO; control), then being treated with androstenone. Culture media and cells were analyzed to assess changes in androstenone metabolism and gene expression. *UGT1A6* was upregulated by treatments targeting both PXR and CAR and downregulated by FXR agonists. Additionally, *PGC1α* and *NR2F1* were downregulated by compounds targeting PXR/CAR, while *FXR* and *NR0B2* were upregulated and *HNF4α* downregulated by treatments acting on FXR. The natural products diallyl sulfide (DAS) and (Z)-guggulsterone (GUG) increased overall androstenone metabolism (DAS, GUG) and the production of Phase I androstenol metabolites (DAS), but only in hepatocyte culture replicates that responded positively to these treatments. Although gene expression was similar between positive-response and negative/non-responsive replicates following treatments, negative/non-responsive replicates for several treatments had higher basal expression of *UGT2B31*, *UGT2A1*, and *SIRT1* and lower basal expression of *FXR*, *PXR*, and *NR0B1* compared to positive-response replicates. These findings suggest that DAS and GUG may be promising treatments for boar taint, specifically in animals with lower basal rates of androstenone metabolism and higher expression of key nuclear receptors.

## Simple Summary

Natural product-derived compounds have been increasingly studied for their potential to treat metabolic diseases. They may also be effective treatments for boar taint, a metabolic issue in intact male pigs (boars), which negatively impacts pork quality. Using cells isolated from boar livers, we found that diallyl sulfide (from garlic) and (Z)-guggulsterone (from the guggul plant) increased the breakdown of androstenone, the primary compound responsible for boar taint. However, these treatments were only effective in a subset of animals. Future feeding trials are warranted to evaluate the efficacy of these natural products as dietary interventions for boar taint and to identify markers that can distinguish animals likely to respond to treatment from those that will not.

## 1. Introduction

The nuclear receptors pregnane X receptor (PXR), constitutive androstane receptor (CAR), and farnesoid X receptor (FXR) are highly expressed in the liver, where they primarily function as transcriptional regulators of bile acid and xenobiotic metabolism [1,2,3]. Beyond these roles, PXR, CAR, and FXR have been shown to regulate the hepatic metabolism of androstenone (5α-androst-16-en-3-one), a 16-androstene pheromone, which is produced in the testis of intact male pigs [4]. Androstenone can accumulate in the adipose tissue and contribute to a meat quality issue known as boar taint, characterized by off-odors or off-flavors in heated pork products. The extent of this accumulation is dependent on the balance between the rate of androstenone synthesis in the testis, metabolism in the liver, and mechanisms that regulate its transport through the circulation and uptake into fat [5,6,7]. Given this complexity, it is difficult to identify which of these pathways is the primary contributor to boar taint in any given animal.

Hepatic androstenone metabolism is a two-phase process. In Phase I, androstenone is reduced to 3β- and 3α-androstenol by the aldo-keto reductases AKR1C1 and AKR1C4, respectively [8]. In Phase II, androstenone and its metabolites are either sulfoconjugated by the cytosolic sulfotransferase enzyme SULT2A1 [9] or glucuronidated by UDP-glucuronosyltransferases (UGTs) [10]. Gray and Squires [4] reported that activation of PXR with rifampicin, FXR with chenodeoxycholic acid (CDCA), and CAR with 6-(4-chlorophenyl)imidazo [2,1-*b*][1,3]thiazole-5-carbaldehyde-O-(3,4-dichlorobenzyl)oxime (CITCO) decreased hepatic expression levels of *AKR1C1* and altered production of 3β-androstenol, with rifampicin-induced activation of PXR significantly increasing the overall metabolism of androstenone in isolated porcine hepatocytes. Additionally, the activation of PXR, CAR, and FXR was shown to increase the hepatic expression of *SULT2A1* and several *UGT1A* and *UGT2B* isoforms to promote bile acid metabolism in both humans and mice, suggesting that these nuclear receptors may also be involved in regulating Phase II androstenone metabolism [11].

While this suggests that compounds targeting PXR, CAR, and FXR could be developed into dietary treatments to increase androstenone metabolism and prevent boar taint, the conventional agonists rifampicin, CITCO, and CDCA are unsuitable due to their potential cytotoxic effects and the risk of drug residues in pork products from treated animals [12,13,14,15,16]. Several bioactive compounds isolated from natural products have been found to regulate the activity of these nuclear receptors in humans and rodents, which may be more suitable for use as dietary interventions to control boar taint. These include hyperforin (HYP), an agonist of PXR found in St. John’s Wort (*Hypericum perforatum*) [17]; diallyl sulfide (DAS), an activator of CAR found in garlic [18,19]; oleanolic acid (OA), a pentacyclic triterpenoid and selective modulator of FXR present in olives (*Olea europaea* L.) and olive oils [20,21]; ginkgolide A (GINK), a potent activator of PXR and CAR found in *Ginkgo biloba* [22,23,24]; and (Z)-guggulsterone (GUG) from the guggul plant (*Commiphora mukul*), which is an agonist of PXR, an inverse agonist of CAR, and an antagonist of FXR [25,26,27].

The objective of this study was to compare the effects of conventional agonists and different natural product treatments on the expression of key genes involved in PXR, CAR, and FXR signaling pathways, as well as the hepatic metabolism of androstenone and resulting metabolite profiles, in isolated porcine hepatocytes from slaughter-weight boars. However, boar taint is a complex trait influenced by various physiological, genetic, nutritional, and environmental factors [25]. As a result, not all animals develop boar taint, and among those that do, not all respond positively to treatment. This inherent biological variability suggests that the effectiveness of these natural product treatments may differ across individual animals. Therefore, this study also assessed differences in androstenone metabolism and gene expression between biological replicates responding positively and those that responded negatively or showed no response to the individual treatments, to investigate possible mechanisms contributing to individual variability in treatment outcomes.

## 2. Materials and Methods

### 2.1. Research Animals and Sample Collection

The present study adhered to the guidelines established by the Canadian Council of Animal Care and the University of Guelph Animal Care Policy regarding the use of animals. Eight terminal cross boars [Duroc × (Landrace × Yorkshire)] were housed at the Arkell Swine Research Facility, University of Guelph. The animals had continuous access to water and were fed standard starter, grower, and finisher rations formulated by Flordale Feed Mill Limited (Floradale, ON, Canada). At an average age of 187.3 ± 6.9 days and weighing approximately 140 kg, the boars were electrically stunned and exsanguinated. Fresh liver lobes were collected for the immediate isolation of primary porcine hepatocytes.

### 2.2. Buffer and Media Preparation

The buffers used for hepatocyte isolation included a blanching buffer (10% Hank’s Balanced Salt Solution (HBSS; 10×, without Ca^2+^, Mg^2+^, HCO_3_^−^, or phenol red), 10 mM HEPES, 1 mM EGTA, pH 7.4), a rinsing buffer (10% HBSS, 10 mM HEPES, pH 7.4), and a digestion buffer (10 mM HEPES, 0.71 mg/mL Type I collagenase in Williams’ Medium E, pH 7.4). Following isolation, hepatocytes were cultured in attachment media (10 mM HEPES, 12.1 nM insulin from bovine pancreas, 10% FBS, and 1% penicillin/streptomycin in Williams’ Medium E, pH 7.4) as well as serum-free media (10 mM HEPES, 12.1 nM insulin from bovine pancreas, 10 mM pyruvate, 0.35 mM L-proline, and 1% penicillin/streptomycin in Williams’ Medium E, pH 7.4).

### 2.3. Isolation of Primary Hepatocytes

The isolation of hepatocytes was performed according to the methods previously described by Bone and Squires [28]. In brief, liver lobes were cannulated and perfused with a blanching solution for 10 min at a flow rate of 25 mL/min. This was followed by a 10 min perfusion with rinsing buffer, and then a digestion buffer was introduced for 30 min at a reduced flow rate of 20 mL/min. After digestion, the liver lobes were carefully dissected to release the hepatocytes. The collected hepatocytes were placed in attachment media, filtered through a 255 µm nylon mesh into 50 mL conical tubes, and centrifuged twice at 100× *g* for 3 min, with the cell pellet washed in between with fresh attachment media and resuspended. Cell viability was assessed using a 0.04% trypan blue exclusion test; if cell viability was 90% or higher, cells were counted with a hemocytometer, resuspended in attachment media, and plated at a density of 0.5 million cells/well in 24-well standard surface-treated polystyrene tissue culture plates (Fisher Scientific, Toronto, ON, Canada). The hepatocytes were incubated for 4 h at 37 °C and 5% CO_2_ in air to allow cell attachment before treatment.

### 2.4. Hepatocyte Treatments

Active compounds from several natural products including diallyl (allyl) sulfide (DAS; ≥97% purity), (Z)-guggulsterone (GUG; ≥95% purity), ginkgolide A (GINK; ≥98% purity), hyperforin (HYP; ≥85% purity), and oleanolic acid (OA; ≥97% purity), as well as conventional agonists 6-(4-chlorophenyl)imidazo[2,1-*b*][1,3]thiazole-5-carbaldehyde-O-(3,4-dichlorobenzyl)oxime (CITCO; ≥98% purity), rifampicin (RIF; ≥97% purity), and chenodeoxycholic acid (CDCA ≥ 96% purity), were purchased from Sigma (Oakville, ON. Canada) and used for hepatocyte treatments. The initial attachment media was replaced with 0.5 mL of serum-free media containing 100 µM DAS, 10 µM GUG, 100 µM GINK, 1 µM HYP, 25 µM OA, 1 µM CITCO, 10 µM RIF, 100 µM CDCA, or 0.05% (*v*/*v*) dimethyl sulfoxide (DMSO) as a vehicle control. Hepatocytes were incubated with these treatments for 20 h, with all treatments performed in triplicate. The media was then replaced with 0.5 mL of serum-free media containing [^3^H]-androstenone (20 µM, 13.5 µCi/µmol, 0.1% ethanol, Moravek Biochemicals Inc., Brea, CA, USA). Hepatocytes were incubated for an additional 3 h, as the rate of androstenone metabolism was previously shown to remain linear up until this time [28]. Following incubation, media and cells were collected to quantify metabolite and gene expression levels, respectively.

### 2.5. Quantification of Androstenone Metabolism and Metabolite Production

Metabolite production was quantified to evaluate the effects of treatments on androstenone metabolism using reverse-phase C18 high-performance liquid chromatography (HPLC). Media collected from hepatocyte incubations were diluted 1:1 (*v*/*v*) with 100% acetonitrile, centrifuged at 8000× *g* for 10 min, and then filtered through a 0.2 µm nylon syringe filter. A 100 µL aliquot was subsequently injected into a Luna 5 µm C18 column (250 × 4.60 mm; Phenomenex, Torrance, CA, USA). Androstenone metabolism was analyzed using a 40 min HPLC profile, as described by Laderoute et al. [9]. The elution of [^3^H] 16-androstene glucuronides, 3α/β-androstenols, and androstenone was monitored with a β-RAM model 2 isotope detector (IN/US Systems, Tampa, FL, USA), with retention times of approximately 3, 33 and 34 min, respectively. Metabolite levels were expressed as a percentage relative to the DMSO control. To assess the impact of individual variability on treatment response, the effect of each natural product and conventional agonist was evaluated based on the observed treatment outcome within each biological replicate. For each treatment, replicates were classified independently as having a positive response (PR) if the disappearance of androstenone, expressed as overall androstenone metabolism, exceeded the percentage observed in the corresponding DMSO control or as negative/non-responsive (NR) if it was less than or equal to this value.

### 2.6. Gene Expression Analysis in Isolated Hepatocytes

For gene expression analysis, hepatocytes were incubated with conventional agonist or natural product treatments for 20 h, as described above, in the absence of androstenone. Following treatment, hepatocytes were collected into 350 µL of lysis buffer, and RNA was extracted using the RNeasy Mini Kit (Qiagen, Santa Clarita, CA, USA) according to the manufacturer’s instructions. The RNA was reverse transcribed, and the resulting cDNA was amplified by Real-Time qPCR (RT-qPCR) as described by Bone and Squires [7]. The genes of interest and their primer sequences are listed in Table 1.

RT-qPCR reactions were performed in duplicate, and the relative fold change for each gene of interest was calculated using β-actin as the housekeeping gene and the DMSO control as the calibrator. Relative fold change values for each gene of interest were standardized by log10 transformation, mean centering, autoscaling, and multiplication by the mean experimental standard deviation as previously described by Willems et al. [29] to minimize variability between biological replicates. For visualization, values were back-transformed using the inverse log10 and then expressed relative to the DMSO control.

### 2.7. Statistical Analysis

The effects of natural products and conventional agonists on androstenone metabolism and gene expression levels in isolated porcine hepatocytes were evaluated using a one-way ANOVA. Post hoc analysis was performed using a significance threshold of *p* ≤ 0.05, and the Benjamini–Hochberg correction was applied to control for multiple comparisons. Statistical analyses were performed in SAS 9.4 (SAS Institute, Cary, NC, USA) using the following model:

y_ijk_ = µ + τ_i_ + ε_ijk_

where y_ij_ represents metabolite or gene expression levels; µ is the overall mean; τ_i_ is the fixed effect of natural product or conventional agonist treatments; and ε_ij_ is the experimental error, which is assumed to have a mean of 0 and a variance of σ^2^.

Differences in hepatic androstenone metabolism and gene expression in response to natural products and conventional agonists were also assessed by treatment response (PR or NR) using a two-way ANOVA with a significance threshold of *p* ≤ 0.05, with the following model:

y_ijk_ = µ + τ_i_ + β_j_ +τβ_ij_ + ε_ijk_

where y_ij_ represents metabolite or gene expression levels; µ is the overall mean; τ_i_ is the fixed effect of treatment (natural product or conventional agonist); β_j_ is the block effect of PR or NR treatment response; τβ_ij_ is the interaction effect between the treatments and blocks; and ε_ij_ is the experimental error, which is assumed to have a mean of 0 and a variance of σ^2^.

Finally, differences in basal metabolite and gene expression levels, measured in DMSO control samples, between PR and NR replicates for each treatment were evaluated using a one-sample *t*-test, testing whether the mean difference (PR-NR) between treatment outcomes was significantly different (*p* ≤ 0.05) from 0.

## 3. Results

### 3.1. Effect of Conventional Agonist Treatments on Hepatic Gene Expression

The expression of genes involved in androstenone metabolism and nuclear receptor signaling was evaluated in isolated porcine hepatocytes treated with RIF, CITCO, and CDCA, conventional agonists of PXR, CAR, and FXR, respectively. Table 2 presents the standardized fold change determined for each gene of interest relative to the DMSO control.

The expression of *AKR1C1*, which mediates the Phase I conversion of androstenone into 3α/β-androstenol, as well as the Phase II genes *SULT2A1*, *UGT1A1*, *UGT1A6*, *UGT2B31*, and *UGT2A1*, was similar to the DMSO control for all three conventional agonist treatments. The most notable effects of RIF, CITCO, and CDCA were observed on the expression of genes encoding nuclear receptors and co-regulatory proteins. *NR0B2* was significantly upregulated by CDCA compared to the DMSO control (*p* = 0.004), and relative to RIF (*p* = 0.002) and CITCO (*p* = 0.004). Additionally, *PGC1α* expression levels were significantly decreased by CITCO (*p* = 0.02) and RIF (*p* = 0.03) compared to the DMSO control but were similar to levels observed with CDCA treatment. No significant differences in the expression levels of other nuclear receptors or co-regulatory proteins were observed across conventional agonist treatments.

### 3.2. Effect of Natural Product Treatments on Hepatic Gene Expression

**Figure 1:**
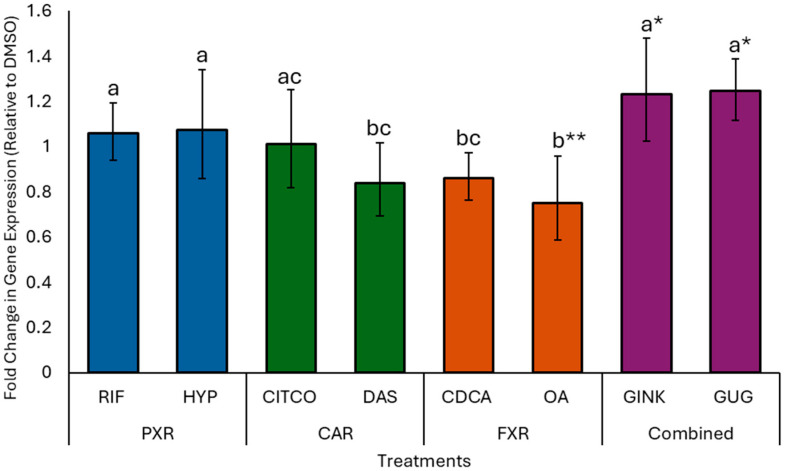
Effects of natural products and conventional treatments on *UGT1A6* expression in isolated hepatocytes, expressed as fold change relative to the dimethyl sulfoxide (DMSO) control. Values are presented as the mean ± 95% CIs for all animals (*n* = 8). Treatments include: rifampicin (RIF) and hyperforin (HYP), which target the pregnane X receptor (PXR); 6-(4-chlorophenyl)imidazo[2,1-*b*][1,3]thiazole-5-carbaldehyde-O-(3,4-dichlorobenzyl)oxime (CITCO) and diallyl sulfide (DAS), which target the constitutive androstane receptor (CAR); chenodeoxycholic acid (CDCA) and oleanolic acid (OA), which target the farnesoid X receptor (FXR); and ginkgolide A (GINK) and (Z)-guggulsterone (GUG), which target multiple receptors (combined receptor targets). Different letters denote significant differences in expression between individual treatments (*p* ≤ 0.05), while differences between individual treatments and the DMSO control are indicated by * (*p* ≤ 0.05) or ** (*p* < 0.01).

Gene expression in response to natural product treatments was evaluated and compared to expression levels following treatment with conventional agonists. Overall, the natural product treatments produced similar gene expression profiles to their corresponding conventional agonist treatment. Expression levels of genes involved in Phase I and II androstenone metabolism were similar to the DMSO control across all natural product treatments, except for *UGT1A6* (Figure 1). Relative to DMSO, *UGT1A6* expression was significantly increased by GINK (*p* = 0.05) and GUG (*p* = 0.04), decreased by OA (*p* = 0.004), and unaffected by DAS and HYP. The expression of *UGT1A6* was similar between GINK, GUG, and HYP, and the conventional agonists RIF and CITCO. *UGT1A6* expression levels were also similar between DAS, OA, and CDCA, but significantly lower than those observed for GINK, GUG, HYP, and RIF.

**Figure 2:**
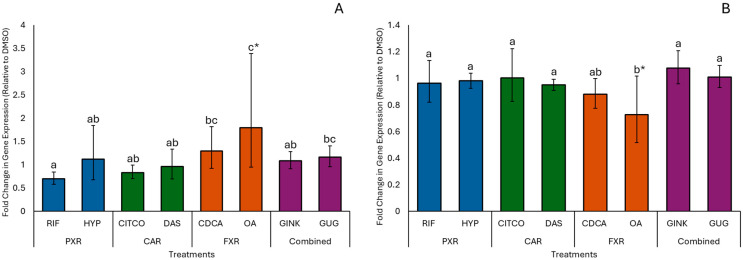
Effects of natural products and conventional treatments on the expression of *FXR* (**A**) and *HNF4α* (**B**) in isolated hepatocytes, expressed as fold change relative to the dimethyl sulfoxide (DMSO) control. Values are presented as the mean ± 95% CIs for all animals (*n* = 8). Treatments include: rifampicin (RIF) and hyperforin (HYP), which target the pregnane X receptor (PXR); 6-(4-chlorophenyl)imidazo[2,1-*b*][1,3]thiazole-5-carbaldehyde-O-(3,4-dichlorobenzyl)oxime (CITCO) and diallyl sulfide (DAS), which target the constitutive androstane receptor (CAR); chenodeoxycholic acid (CDCA) and oleanolic acid (OA), which target the farnesoid X receptor (FXR); and ginkgolide A (GINK) and (Z)-guggulsterone (GUG), which target multiple receptors (combined receptor targets). Different letters denote significant differences in expression between individual treatments (*p* ≤ 0.05), while differences between individual treatments and the DMSO control are indicated by * (*p* ≤ 0.05).

Natural product treatments also affected the expression of genes for several nuclear receptors and co-regulatory proteins. Treatment with OA significantly increased *FXR* expression (*p* = 0.02; Figure 2A) and decreased *HNF4α* expression (*p* = 0.008; Figure 2B) relative to the DMSO control. Other natural products and conventional agonists did not significantly affect the expression of these genes compared to DMSO. However, expression levels of *FXR* and *HNF4α* were similar between OA and CDCA.

**Figure 3:**
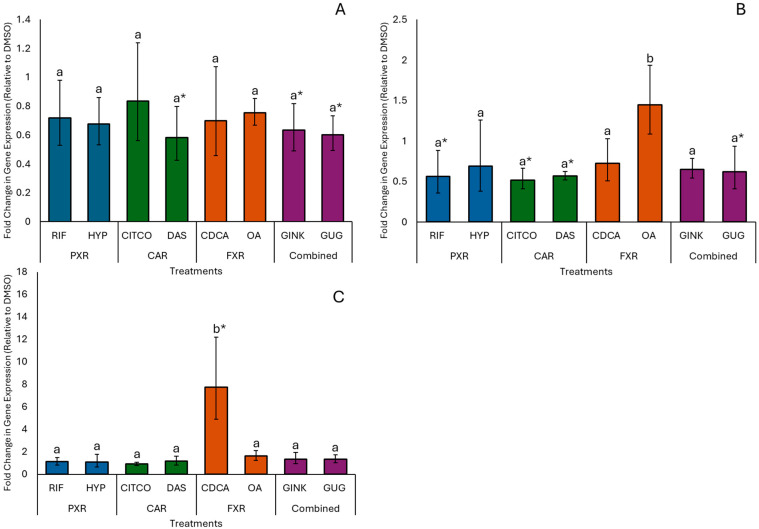
Effects of natural products and conventional treatments on the expression of *NR2F1* (**A**), *PGC1α* (**B**), and *NR0B2* (**C**) in isolated hepatocytes, expressed as fold change relative to the dimethyl sulfoxide (DMSO) control. Values are presented as the mean ± 95% CIs for all animals (*n* = 8). Treatments include: rifampicin (RIF) and hyperforin (HYP), which target the pregnane X receptor (PXR); 6-(4-chlorophenyl)imidazo[2,1-*b*][1,3]thiazole-5-carbaldehyde-O-(3,4-dichlorobenzyl)oxime (CITCO) and diallyl sulfide (DAS), which target the constitutive androstane receptor (CAR); chenodeoxycholic acid (CDCA) and oleanolic acid (OA), which target the farnesoid X receptor (FXR); and ginkgolide A (GINK) and (Z)-guggulsterone (GUG), which target multiple receptors (combined receptor targets). Different letters denote significant differences in expression between individual treatments (*p* ≤ 0.05), while differences between individual treatments and the DMSO control are indicated by * (*p* ≤ 0.05).

Significant downregulation of *NR2F1* (Figure 3A) by DAS (*p* = 0.03), GINK (*p* = 0.03), and GUG (*p* = 0.02) and *PGC1α* (Figure 3B) by DAS (*p* = 0.03) and GUG (*p* = 0.05) was observed relative to DMSO. Expression levels of both genes following treatment with other natural products and conventional agonists were similar to those observed with DAS, GINK, and GUG, except for OA, which significantly increased *PGC1α* expression relative to all other treatments, but not DMSO. A similar expression pattern was observed for *NR0B2* (Figure 3C), which was significantly upregulated by CDCA but not by other natural products or conventional agonists.

### 3.3. Effect of Treatments on Androstenone Metabolism by Isolated Hepatocytes

To investigate the effects of natural products and conventional agonists on hepatic androstenone metabolism, we quantified overall androstenone metabolism from the decrease in the initial androstenone concentration, along with the production of Phase I 3α/β-androstenols and Phase II 16-androstene glucuronides, by isolated hepatocytes in response to each treatment. Treatment with DAS significantly increased (*p* = 0.0008) the production of 3α/β-androstenols relative to the DMSO control, with levels reaching 266.2 ± 74.3% of the control. In contrast, both overall androstenone metabolism and 16-androstene glucuronide production were unaffected by natural product and conventional agonist treatments. However, individual treatment responses varied across biological replicates of hepatocyte cultures. Relative to DMSO, DAS and RIF increased overall androstenone metabolism in 75% of replicates, GINK, GUG, and HYP in 63%, CITCO in 50%, OA in 37.5%, and CDCA in 25%, suggesting that the effects of these treatments on hepatic androstenone metabolism and related gene expression may differ by treatment outcome.

### 3.4. Differences in Androstenone Metabolism and Gene Expression by Treatment Response

**Figure 4:**
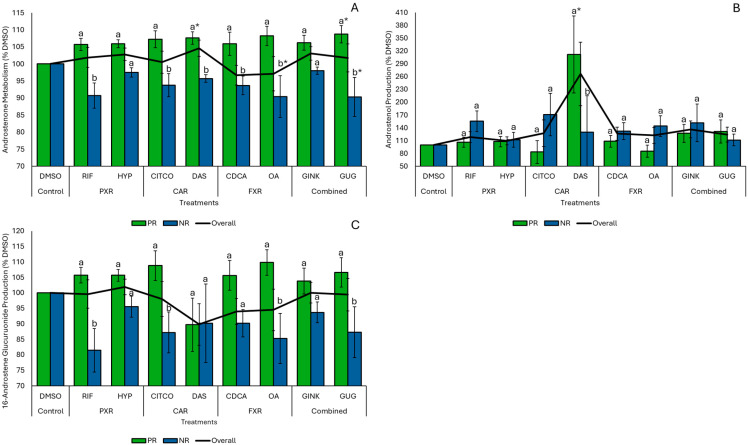
Effects of natural products and conventional treatments on overall androstenone metabolism (**A**), the production of 3α/β-androstenol (**B**), and the production of 16-androstene glucuronides (**C**) by isolated hepatocytes, expressed as a percentage relative to the dimethyl sulfoxide (DMSO) control. Values are presented as the mean ± SE for all animals (overall; *n* = 8) and for subsets of biological replicates classified as having a positive response (PR) or as negative/non-responsive (NR) to individual treatments. Treatments include: rifampicin (RIF) and hyperforin (HYP), which target the pregnane X receptor (PXR); 6-(4-chlorophenyl)imidazo[2,1-*b*][1,3]thiazole-5-carbaldehyde-O-(3,4-dichlorobenzyl)oxime (CITCO) and diallyl sulfide (DAS), which target the constitutive androstane receptor (CAR); chenodeoxycholic acid (CDCA) and oleanolic acid (OA), which target the farnesoid X receptor (FXR); and ginkgolide A (GINK) and (Z)-guggulsterone (GUG), which target multiple receptors (combined receptor targets). Statistically significant differences (*p* ≤ 0.05) between treatments and the DMSO control are indicated by (*), while differences between PR and NR replicates within the same treatment are denoted by different letters.

To account for variability associated with treatment outcomes, responses in each biological replicate were classified separately for each treatment as positive (PR) if overall androstenone metabolism exceeded the corresponding DMSO control for a given biological replicate, or negative/non-responsive (NR) if it was less than or equal to the control. The overlap in PR classification between natural product and conventional agonist treatments ranged from 50% to 100%. Overall androstenone metabolism was significantly greater in PR replicates compared to NR replicates for all treatments except GINK and HYP (Figure 4A). In PR replicates, overall androstenone metabolism was significantly increased by DAS (*p* = 0.04) and GUG (*p* = 0.05) relative to the DMSO control and decreased by GUG (*p* = 0.04) and OA (*p* = 0.05) in NR replicates. The production of 3α/β-androstenol was also significantly greater in PR replicates compared to both NR replicates (*p* = 0.007) and the DMSO control (p = 0.002) following DAS treatment (Figure 4B). However, 3α/β-androstenol production was unaffected by other natural products and conventional agonists, and similar production levels were observed between PR and NR replicates. Treatment with GUG (*p* = 0.02), OA (*p* = 0.02), CITCO (*p* = 0.02), and RIF (*p* = 0.02) significantly increased the production of 16-androstene glucuronides in PR compared to NR replicates (Figure 4C); however, levels among PR and NR replicates remained comparable to the DMSO control across all treatments.

While PR and NR replicates showed clear differences in overall androstenone metabolism and metabolite production in response to both natural product and conventional agonist treatments, gene expression patterns were unexpectedly similar across the two groups. Therefore, we evaluated whether basal metabolite and gene expression levels differed between PR and NR replicates for each treatment. Table 3 shows the mean difference (PR-NR) in (A) basal metabolite levels, quantified as percent abundance in DMSO control samples, and (B) basal gene expression, measured as fold change relative to β-actin in DMSO controls, for the transcripts of interest.

Basal 3α/β-androstenol production was significantly greater (*p* = 0.00009) in NR than in PR replicates of DAS, and numerically, but not significantly higher in NR replicates for other treatments. Additionally, basal expression levels of several genes were significantly higher in NR than in PR replicates of specific treatments, including *UGT2B31* for DAS (*p* = 0.007) and CITCO (*p* = 0.04), *UGT2A1* for OA (*p* = 0.04), and *SIRT1* for DAS (*p* = 0.03), GINK (*p* = 0.02), HYP (*p* = 0.02), and CITCO (*p* = 0.01). For other treatments, NR replicates consistently had numerically, but not significantly, higher basal expression of these genes. In contrast, PR replicates of CITCO, GUG, and RIF had significantly greater basal expression of *FXR* (*p* = 0.05), *PXR* (*p* = 0.04), and *NR0B1* (*p* = 0.01), respectively, than NR replicates for these treatments, with basal expression of *FXR*, *PXR*, *UGT1A1*, and *HNF1α* also being numerically, but not significantly, higher in PR replicates across all treatments. These differences in basal gene expression indicate that gene expression levels were highly variable between individual animals, which may partially explain why differences in androstenone metabolism and metabolite production observed between PR and NR replicates were not associated with post-treatment changes in gene expression.

## 4. Discussion

In recent years, bioactive compounds isolated from natural products that target the nuclear receptors PXR, CAR, and FXR have been increasingly studied as drug candidates for the management of metabolic disorders [30,31,32]. In this study, we evaluated the natural product-derived compounds DAS, GINK, GUG, HYP, and OA as potential treatments for boar taint, a meat quality issue that develops in intact male pigs. Specifically, we compared their effects on hepatic androstenone metabolism and the expression of related genes in primary porcine hepatocytes isolated from slaughter-weight boars, relative to those of conventional PXR, CAR, and FXR agonists, RIF, CITCO, and CDCA, respectively.

RIF, CITCO, and CDCA were previously reported to modulate androstenone metabolism in isolated porcine hepatocytes [4], where all three compounds were shown to downregulate *AKR1C1*. However, in the present study, these conventional agonists had no significant effect on the expression of genes involved in Phase I and II androstenone metabolism. In contrast, *UGT1A6*, a Phase II gene involved in the glucuronidation of endogenous and exogenous compounds [33,34], was upregulated by GINK and GUG, natural products that target both PXR and CAR, and downregulated by OA, a selective FXR modulator. Since *UGT1A6* expression was unaffected by treatments targeting PXR (RIF, HYP) or CAR (CITCO, DAS) alone, our results suggest that Phase II androstenone metabolism in boars may be influenced by crosstalk between PXR and CAR signaling pathways. Supporting this, both PXR and CAR were shown to regulate *UGT1A6* transcription in mice [35], and increased UGT1A6 activity was reported in primary human hepatocyte cultures following treatment with carbamazepine, a dual activator of PXR and CAR [36,37,38].

Crosstalk among PXR, CAR, FXR, and other nuclear receptors or co-regulatory proteins is well established and plays an important role in regulating metabolism [39,40], and we have previously summarized the potential signaling pathways and co-regulatory proteins involved in regulating androstenone metabolism in a recent review [16]. PGC1α is a common coactivator and transcriptional regulator of PXR and CAR, while NR2F1 has been shown to inhibit PXR activity by competing for DNA response element binding [41,42,43]. Additionally, FXR and HNF4α share approximately 50% of the same DNA binding sites in mouse liver and their cooperative activity was shown to regulate the transcription of shared target genes, including the transcriptional repressor *NR0B2* [44,45]. In the present study, *PGC1α* was downregulated by treatments targeting PXR (RIF), CAR (DAS, CITCO), or both PXR and CAR (GUG), and *NR2F1* was downregulated by the natural products DAS, GINK, and GUG. In contrast, treatments targeting FXR (CDCA, OA) increased the expression of *FXR* (OA) and *NR0B2* (CDCA) and decreased *HNF4α* expression (OA). Together, these findings suggest that the metabolic effects of natural products and conventional agonists in boars are influenced by complex interactions between PXR, CAR, and FXR, as well as other nuclear receptors and co-regulatory proteins.

Previous work by Gray and Squires [4] demonstrated that 3β-androstenol production was increased by CITCO, and overall androstenone metabolism increased by RIF. In contrast, we found no significant effects of the conventional agonists CITCO, CDCA, and RIF on androstenone metabolism or metabolite production. However, DAS significantly increased 3α/β-androstenol production, consistent with previously reported effects of the corresponding conventional CAR agonist, CITCO [4]. The discrepancies between our findings and those of Gray and Squires [4] may reflect differences in the basal activity of the hepatocyte culture systems, as the breed of boars used and the incubation duration for androstenone metabolism (3 h in the present study vs. 20 h previously) differed between studies. A higher basal rate of androstenone metabolism in our culture system may also explain why treatment-induced changes in gene expression did not correspond to overall changes in androstenone metabolism or metabolite production. The system may have been operating near capacity, limiting the extent to which changes in gene expression could further increase enzyme activity to produce measurable effects on androstenone metabolism. However, further work using receptor-specific inhibitors or directly measuring basal enzyme activity is required to confirm this.

Individual variability in treatment response may have further contributed to the limited effects observed, with treatments targeting PXR and CAR increasing overall androstenone metabolism in 50% to 75% of biological replicates of hepatocyte cultures, and those targeting FXR in 25% to 37.5% of replicates. To further investigate this, we examined the effects of each natural product and conventional agonist individually within each biological replicate, comparing replicates with increased (PR) overall androstenone metabolism to those that showed decreased or unchanged (NR) metabolism relative to the control, for each treatment. Overall androstenone metabolism and 16-androstene glucuronide production were greater in PR compared to NR replicates for most treatments. Both DAS and GUG increased overall androstenone metabolism relative to the control in PR replicates, with DAS causing a significant increase in 3α/β-androstenol production within PR replicates compared to NR, and GUG promoting slight, non-significant increases to both Phase I and II metabolite levels in PR replicates. Interestingly, basal production of 3α/β-androstenol was significantly higher in NR compared to PR replicates of DAS, suggesting that treatment effects may be greater in animals with lower basal rates of androstenone metabolism.

Treatment-specific differences were also observed in the basal expression of several genes, many of which are involved in Phase II metabolism or encode key nuclear receptors. Compared to PR replicates, the basal expression of *UGT2B31* was higher in NR replicates of DAS and CITCO, and the basal expression of *UGT2A1* was higher in NR replicates of OA. In contrast, the basal expression of *FXR*, *PXR*, and *NR0B1* was greater in PR than in NR replicates of CITCO, GUG, and RIF, respectively. Following treatment, expression levels of these genes were similar between PR and NR replicates. The considerable individual variability in basal gene expression may have masked treatment-induced changes in gene expression, resulting in similar expression levels between PR and NR replicates following treatment, despite observed differences in androstenone metabolism and metabolite production. Furthermore, the observed differences in basal gene expression between PR and NR replicates suggest that variation in treatment response may result from pre-existing differences in the expression of UGTs and key nuclear receptors, rather than from divergent effects of the treatments themselves. These results demonstrate the importance of Phase II metabolism and nuclear receptor availability in influencing individual responses to natural products and conventional agonists.

Consistent with our results, significant interindividual variability in the hepatic expression of *PXR* and *CAR* has been reported in humans [46,47], and CAR mRNA levels are known to oscillate throughout the day under the control of the circadian rhythm [48]. The effects of GUG in mice have also been shown to depend on the CAR to PXR ratio [26], further highlighting the importance of nuclear receptor availability. Additionally, individual differences in basal rates of androstenone metabolism may reflect further variation in hepatic xenobiotic metabolism, which could affect the conversion of natural product parent compounds to their bioactive metabolites to influence treatment response. For example, the metabolism of GUG by human liver microsomes was shown to produce four distinct metabolites, which differed from GUG in their induction strength for shared target genes [49]. Similarly, both DAS and its metabolite, allyl methyl sulfide, were reported to reduce protein levels of the cytochrome P450 enzyme CYP2E1 without altering its mRNA expression, with DAS exhibiting a stronger inhibitory potency [50,51]. Since CYP2E1 is a major Phase I enzyme responsible for the metabolism of skatole, a microbial metabolite that also contributes to boar taint [52], future research should also evaluate the effects of natural products on hepatic skatole metabolism.

While our results suggest that DAS and GUG may be promising dietary treatments for boar taint, treatment response was variable across individual animals. In this study, phenotypes (PR and NR) were defined based on the effects of individual treatments on overall androstenone metabolism relative to the control. However, overall androstenone metabolism is a complex process regulated by the coordinated action of multiple genes and enzymes involved in Phase I and II pathways. Furthermore, several physiological processes beyond hepatic metabolism also influence androstenone accumulation in fat, including the rate of androstenone synthesis in the testis, its transport through the circulation, and the mechanisms regulating its uptake into adipose tissue. These processes could not be measured or accounted for with the model system used in the present study. Therefore, future in vivo feeding trials are needed to evaluate the effect of DAS and GUG on boar taint levels to provide a more physiologically relevant measure of treatment efficacy. These feeding trials also present an opportunity to identify biological and genetic markers associated with variation in treatment response, which could improve the accuracy of evaluating and predicting the effectiveness of these treatments for controlling boar taint.

## 5. Conclusions

This study demonstrated that natural products and their corresponding conventional agonist treatments produce similar effects on androstenone metabolism and the expression of related genes in isolated hepatocytes from boars. The natural products DAS and GUG increased hepatic androstenone metabolism in a subset of boars responding positively to these treatments. However, treatment response varied across individual animals and appears to be influenced by several factors, including the individual’s basal rate of androstenone metabolism, the availability of key nuclear receptors, and crosstalk between nuclear receptors and co-regulatory proteins. Further research is needed to investigate the effects of natural product treatments on hepatic skatole metabolism. In addition, in vivo feeding trials with DAS and GUG are necessary to assess their effects on boar taint levels and to identify the biological and genetic markers associated with treatment response.

## Figures and Tables

**Table 1:** Gene names, abbreviations, primer sequences, and NCBI reference sequences (RefSeq ID) for gene transcripts of interest.

Gene Name	Abbreviation	Primer Forward Sequence (5′-3′)	Primer Reverse Sequence (5′-3′)	Refseq ID
Aldo-keto reductase 1C1	*AKR1C1*	GGAGGACTTTTTCCCAAAGG	TCCCTCGTTCTTGCACTTCT	NM_001044618
Sulfotransferase 2A1	*SULT2A1*	ACACGAGAAGCGCCGTAGAG	TGGACATGTTGTTTTCTTTCATGA	NM_001037150
UDP-glucuronosyltransferase 1A6	*UGT1A6*	TGCTTTGGGCAAAATACCTC	CTTTGGGTGACCAAGCAGAT	NM_001278750.1
UDP-glucuronosyltransferase 1A1	*UGT1A1*	ATAATTACCCGAGGCCCATC	CCCCAAAGAGAAAACCACAA	KJ922612.1
UDP-glucuronosyltransferase 2A1	*UGT2B31*	TTTGAGACAATGGGGAAAGC	AGGTAGGGGTTTTGCAGGTT	XM_003356958.4
UDP-glucuronosyltransferase 2B31	*UGT2A1*	TGCACGTTACTGAAAATGCAAG	TTGTAAAAGCCAGAGCACATCA	NM_001244124.1
Farnesoid X receptor	*FXR*	GTCGTCAAGGGAAGAAGCTG	TTTCCCACTGTTGTCTGCTG	NM_001038005
Pregnane X receptor	*PXR*	GCCATCTCCCTTTTCTCTCC	CGATGTAGGCCTTCAGGGTA	NM_001038005
Constitutive androstane receptor	*CAR*	CCGCCATATGGGCACTATGT	GCGAAATGCATGAGCAGAGA	NM_001037996
Nuclear receptor subfamily 0 group B member 2	*NR0B2*	AGTGCTGCCTGGAGTCCTTA	GATGTGGGAGGAGGCATAGA	XM_003127720.4
Nuclear receptor subfamily 2 group F member 1	*NR2F1*	ACAGGAACTGTCCCATCGAC	GATGTAGCCGGACAGGTAGC	XM_003354213.4
Hepatocyte nuclear factor 4 alpha	*HNF4α*	ACCTCCCCTGTCTCTGGAAT	ATGTACTTGGCCCACTCGAC	NM_001044571.1
Hepatocyte nuclear factor 1 alpha	*HNF1α*	CCATCCTCAAAGAGCTGGAG	GCTGCAGGTAGGACTTGACC	NM_001032388.1
Peroxisome proliferator-activated receptor gamma coactivator 1-alpha	*PGC1α*	TAAAGATGCCGCCTCTGACT	TGACCGAAGTGCTTGTTCAG	NM_213963.2
NAD-dependent deacetylase sirtuin-1	*SIRT1*	CCATGGCGCTGAGGTATATT	TCATCCTCCATGGGTTCTTC	NM_001145750.2
Steroid receptor coactivator 1	*SRC1*	AGTGATGACTCGTGGCACTG	GCTGCATGTCTGGACTTTGA	NM_001025228.1
High mobility group box 1 protein	*HMGB1*	CCATTGGTGATGTTGCAAAG	CCCTTTAGCTCGGTATGCAG	NM_001004034.1
β-Actin	-	CGTGGACATCAGGAAGGAC	TCTGCTGGAAGGTGGACAG	XM_003357928

**Table 2:** Expression of genes involved in hepatic androstenone metabolism and nuclear receptor signaling.

		Treatment		
	Transcript	CITCO	CDCA	RIF
Phase I metabolism	*AKR1C1*	0.93 (0.72, 1.20)	0.90 (0.60, 1.35)	0.95 (0.86, 1.06)
Phase II metabolism	*SULT2A1*	0.73 (0.23, 2.28)	4.01 (0.74, 21.75)	1.04 (0.45, 2.40)
	*UGT1A1*	0.82 (0.54, 1.25)	0.73 (0.47, 1.12)	1.14 (0.86, 1.51)
	*UGT1A6*	1.01 (0.82, 1.25) ^ab^	0.86 (0.76, 0.97) ^b^	1.06 (0.94, 1.19) ^a^
	*UGT2B31*	1.07 (0.84, 1.36)	1.01 (0.84, 1.21)	1.02 (0.82, 1.27)
	*UGT2A1*	0.29 (0.09, 0.93)	0.52 (0.17, 1.64)	0.30 (0.10, 0.86)
Nuclear receptors	*FXR*	0.84 (0.70, 0.99) ^a^	1.29 (0.92, 1.82) ^a^	0.70 (0.58, 0.85) ^b^
	*PXR*	0.84 (0.73, 0.96) ^ab^	1.30 (1.01, 1.67) ^a^	0.78 (0.71, 0.85) ^b^
	*CAR*	0.93 (0.76, 1.14)	0.97 (0.89, 1.06)	0.78 (0.63, 0.96)
	*NR0B2*	0.92 (0.79, 1.07) ^a^	7.74 (4.90, 12.21) **^b^	1.11 (0.83, 1.47) ^a^
	*NR2F1*	0.83 (0.56, 1.24)	0.70 (0.46, 1.07)	0.72 (0.53, 0.98)
	*HNF1α*	4.62 (3.01, 7.09)	3.58 (1.45, 8.81)	5.77 (1.48, 22.48)
	*HNF4α*	1.00 (0.83, 1.22)	0.88 (0.78, 1.00)	0.96 (0.82, 1.13)
Co-regulatory proteins	*PGC1α*	0.52 (0.41, 0.66) *	0.72 (0.51, 1.03)	0.56 (0.36, 0.89) *
	*SIRT1*	0.88 (0.73, 1.06)	0.83 (0.68, 1.02)	0.81 (0.75, 0.87)
	*SRC1*	0.91 (0.82, 1.03)	0.91 (0.81, 1.01)	0.85 (0.81, 0.88)
	*HMGB1*	0.83 (0.61, 1.11)	0.81 (0.55, 1.18)	0.83 (0.77, 0.91)

Values are expressed as the mean standardized fold change with 95% CIs relative to the dimethyl sulfoxide (DMSO) control (*n* = 8). Treatments include: 6-(4-chlorophenyl)imidazo[2,1-*b*][1,3]thiazole-5-carbaldehyde-O-(3,4-dichlorobenzyl)oxime (CITCO), chenodeoxycholic acid (CDCA), and rifampicin (RIF). Significant differences in gene expression between a given treatment and the DMSO control are indicated by * *p* ≤ 0.05 or ** *p* < 0.01. Different letters within a row indicate significant differences (*p* ≤ 0.05) in the expression of the corresponding gene between treatment groups.

**Table 3:** Differences in basal metabolite (**A**) and gene expression (**B**) levels between positive and negative/non-responsive hepatocyte replicates by treatment.

**(A)**			**Basal Metabolite Abundance Difference (PR-NR, %)**					
**Receptor**	**Treatments**		**Androstenone Metabolism**		**3α/β-Androstenols**		**16-Androstene Glucuronides**	
PXR	RIF		30.99 ± 18.38		−2.77 ± 2.88		14.75 ± 11.21	
	HYP		−9.45 ± 5.10		−3.31 ± 2.90		−6.96 ± 6.06	
CAR	CITCO		2.30 ± 7.44		−2.84 ± 2.17		4.53 ± 7.27	
	DAS		−8.51 ± 4.56		−6.59 ± 0.58 ***		−1.51 ± 4.86	
FXR	CDCA		6.32 ± 4.56		−0.95 ± 1.55		6.86 ± 4.55	
	OA		0.23 ± 6.27		−2.75 ± 1.75		2.49 ± 6.13	
Combined	GINK		−9.45 ± 5.10		−3.31 ± 2.90		−6.96 ± 6.06	
	GUG		5.10 ± 9.79		−5.00 ± 1.91		10.58 ± 8.10	
**(B)**	**Basal Expression Difference (PR-NR, ΔCT)**							
**Transcript**	**PXR**		**CAR**		**FXR**		**Combined**	
	**RIF**	**HYP**	**CITCO**	**DAS**	**CDCA**	**OA**	**GINK**	**GUG**
*AKR1C1*	−1.66 ± 1.02	−0.17 ± 1.21	−0.35 ± 1.16	−0.48 ± 1.79	0.17 ± 0.62	0.91 ± 0.86	−0.17 ± 1.21	−0.58 ± 1.18
*SULT2A1*	0.11 ± 0.45	−1.00 ± 0.67	−0.52 ± 0.90	−0.42 ± 0.66	−0.54 ± 0.45	0.48 ± 0.94	−1.00 ± 0.67	0.13 ± 0.79
*UGT1A1*	−0.26 ± 0.64	−0.50 ± 0.56	−0.90 ± 0.57	−0.20 ± 0.62	−1.44 ± 0.25	−0.53 ± 0.91	−0.50 ± 0.56	−0.57 ± 0.56
*UGT1A6*	−0.079 ± 0.61	0.014 ± 0.42	−0.35 ± 0.43	0.030 ± 0.54	−0.23 ± 0.23	0.15 ± 0.41	0.014 ± 0.42	−0.34 ± 0.43
*UGT2B31*	0.08 ± 0.98	1.05 ± 0.49	0.55 ± 0.62 *	1.35 ± 0.26 **	0.93 ± 0.33	1.03 ± 0.36	1.05 ± 0.49	0.62 ± 0.68
*UGT2A1*	0.076 ± 0.90	0.79 ± 0.45	0.33 ± 0.60	0.66 ± 0.54	0.84 ± 0.29	0.93 ± 0.31 *	0.79 ± 0.45	0.086 ± 0.61
*FXR*	−0.79 ± 0.24	−0.30 ± 0.35	−0.73 ± 0.26 *	−0.23 ± 0.34	−0.39 ± 0.21	−0.33 ± 0.28	−0.30 ± 0.35	−0.62 ± 0.31
*PXR*	−1.01 ± 0.32	−0.28 ± 0.45	−0.85 ± 0.35	−0.45 ± 0.34	−0.43 ± 0.27	−0.34 ± 0.35	−0.28 ± 0.45	−0.95 ± 0.30 *
*CAR*	−0.12 ± 0.27	0.071 ± 0.35	−0.012 ± 0.49	0.0030 ± 0.27	−0.86 ± 0.17	0.12 ± 0.83	0.071 ± 0.35	−0.080 ± 0.35
*NR0B2*	−1.46 ± 0.32 *	0.36 ± 0.79	−0.21 ± 0.72	−0.015 ± 1.15	0.88 ± 0.38	0.28 ± 0.72	0.36 ± 0.79	−0.59 ± 0.79
*NR2F1*	0.13 ± 0.26	0.0075 ± 0.32	0.11 ± 0.45	0.065 ± 0.25	−0.32 ± 0.19	−0.52 ± 0.22	0.0075 ± 0.32	0.16 ± 0.32
*HNF1α*	−3.32 ± 1.25	−0.46 ± 2.12	−2.10 ± 2.18	−2.18 ± 1.40	−3.13 ± 1.09	−0.12 ± 2.87	−0.46 ± 2.12	−3.46 ± 1.43
*HNF4α*	−0.39 ± 0.85	−0.45 ± 0.49	−0.13 ± 0.43	−0.51 ± 0.77	0.37 ± 0.25	−0.14 ± 0.38	−0.45 ± 0.49	−0.10 ± 0.55
*PGC1α*	0.20 ± 0.29	−0.11 ± 0.46	−0.12 ± 0.35	0.53 ± 0.23	−0.34 ± 0.20	−0.039 ± 0.36	−0.11 ± 0.46	0.44 ± 0.31
*SIRT1*	0.27 ± 0.25	0.59 ± 0.12 *	0.59 ± 0.13 *	0.48 ± 0.15 *	0.18 ± 0.15	0.35 ± 0.19	0.59 ± 0.12 *	0.41 ± 0.21
*SRC1*	0.018 ± 0.47	0.39 ± 0.24	0.14 ± 0.33	0.46 ± 0.21	−0.17 ± 0.17	0.33 ± 0.41	0.39 ± 0.24	0.13 ± 0.32
*HMGB1*	−0.44 ± 0.20	0.36 ± 0.32	0.16 ± 0.35	0.054 ± 0.38	0.59 ± 0.16	0.47 ± 0.25	0.36 ± 0.32	−0.16 ± 0.33

Values are expressed as the mean ± SE of the percent difference in metabolite levels (A) and the mean ± SE of the difference in ΔCT values (B) measured in dimethyl sulfoxide (DMSO) control incubations between positive response (PR) and negative/non-responsive (NR) hepatocyte replicates for each treatment. Treatments include: rifampicin (RIF) and hyperforin (HYP), which target the pregnane X receptor (PXR); 6-(4-chlorophenyl)imidazo[2,1-*b*][1,3]thiazole-5-carbaldehyde-O-(3,4-dichlorobenzyl)oxime (CITCO) and diallyl sulfide (DAS), which target the constitutive androstane receptor (CAR); chenodeoxycholic acid (CDCA) and oleanolic acid (OA), which target the farnesoid X receptor (FXR); and ginkgolide A (GINK) and (Z)-guggulsterone (GUG), which target multiple receptors (combined receptor targets). Values that differ significantly from 0 are indicated by * (*p* ≤ 0.05), ** (*p* < 0.01), or *** (*p* < 0.0001).

## Data Availability

The original contributions presented in the study are included in the article, further inquiries can be directed to the corresponding author.

## Abbreviations

The following abbreviations are used in this manuscript:

PXR	Pregnane X receptor
CAR	Constitutive androstane receptor
FXR	Farnesoid X receptor
UGTs	UDP-Glucuronosyltransferases
RIF	Rifampicin
CDCA	Chenodeoxycholic acid
CITCO	6-(4-chlorophenyl)imidazo[2,1-*b*][1,3]thiazole-5-carbaldehyde-O-(3,4-dichlorobenzyl)oxime
HYP	Hyperforin
DAS	Diallyl sulfide
OA	Oleanolic acid
GINK	Ginkgolide A
GUG	(Z)-Guggulsterone
HBSS	Hank’s Balanced Salt Solution
DMSO	Dimethyl sulfoxide
HPLC	High-performance liquid chromatography
PR	Positive response
NR	Negative/non-responsive
RT-qPCR	Real-Time Quantitative Polymerase Chain Reaction
AKR1C1	Aldo-keto reductase 1C1
SULT2A1	Sulfotransferase 2A1
UGT1A6	UDP-glucuronosyltransferase 1A6
UGT1A1	UDP-glucuronosyltransferase 1A1
UGT2A1	UDP-glucuronosyltransferase 2A1
UGT2B31	UDP-glucuronosyltransferase 2B31
NR0B2	Nuclear receptor subfamily 0 group B member 2
NR2F1	Nuclear receptor subfamily 2 group F member 1
HNF4α	Hepatocyte nuclear factor 4 alpha
HNF1α	Hepatocyte nuclear factor 1 alpha
PGC1α	Peroxisome proliferator-activated receptor gamma coactivator 1-alpha
SIRT1	NAD-dependent deacetylase sirtuin-1
SRC1	Steroid receptor coactivator 1
HMGB1	High mobility group box 1 protein
CYP2E1	Cytochrome P450 2E1
